# Generating Ensembles of Dynamic Misfolding Proteins

**DOI:** 10.3389/fnins.2022.881534

**Published:** 2022-03-31

**Authors:** Theodoros K. Karamanos, Arnout P. Kalverda, Sheena E. Radford

**Affiliations:** Astbury Centre for Structural Molecular Biology, School of Molecular and Cellular Biology, University of Leeds, Leeds, United Kingdom

**Keywords:** ensemble calculations, protein misfolding, machine learning, intrinsic disorder, oligomerization, NMR spectroscopy

## Abstract

The early stages of protein misfolding and aggregation involve disordered and partially folded protein conformers that contain a high degree of dynamic disorder. These dynamic species may undergo large-scale intra-molecular motions of intrinsically disordered protein (IDP) precursors, or flexible, low affinity inter-molecular binding in oligomeric assemblies. In both cases, generating atomic level visualization of the interconverting species that captures the conformations explored and their physico-chemical properties remains hugely challenging. How specific sub-ensembles of conformers that are on-pathway to aggregation into amyloid can be identified from their aggregation-resilient counterparts within these large heterogenous pools of rapidly moving molecules represents an additional level of complexity. Here, we describe current experimental and computational approaches designed to capture the dynamic nature of the early stages of protein misfolding and aggregation, and discuss potential challenges in describing these species because of the ensemble averaging of experimental restraints that arise from motions on the millisecond timescale. We give a perspective of how machine learning methods can be used to extract aggregation-relevant sub-ensembles and provide two examples of such an approach in which specific interactions of defined species within the dynamic ensembles of α-synuclein (αSyn) and β_2_-microgloblulin (β_2_m) can be captured and investigated.

## Introduction

Although significant recent progress in computational methods has enabled the prediction of the native structure of a protein and of protein complexes given primary sequence information alone (Yang et al., 2020; Jumper et al., 2021), understanding how a protein misfolds and defining the structural properties of misfolded and aberrantly assembled/aggregated species remain largely a mystery. Protein misfolding represents a critical missing link in our knowledge of protein chemistry as it is represents a fundamental property of the polypeptide chain and is directly linked with numerous human disorders including neurodegeneration, cataract formation, type II diabetes mellitus (Knowles et al., 2014; Chiti and Dobson, 2017; Iadanza et al., 2018; Sawaya et al., 2021). More than 40 proteins has been identified as the culprits of aggregation in human amyloid diseases (Benson et al., 2020). Pathological protein self-assembly reactions do not only result in highly ordered amyloid fibrils but also in the formation of amorphous aggregates that lack long range order or a common underlying structure, misfolded oligomers, or phase-separated protein condensates (Ebo et al., 2020; Mathieu et al., 2020). In this review the term “aggregation” largely refers to protein polymerization on pathway to amyloid unless otherwise stated.

**FIGURE 1:**
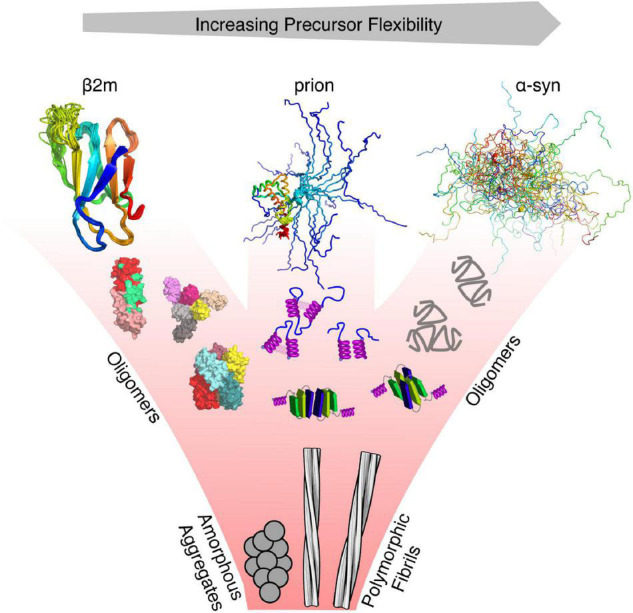
Protein misfolding and flexibility. Examples of proteins with different degrees of flexibility (α-synuclein—IDP, prion protein –folded and IDR, and β_2_m—folded), each of which aggregate to form amyloid fibrils. For each class of protein, its structure cannot be represented by a single conformation, as each interconverts between various conformers on different timescales. Hence, the conformational properties of these proteins are best described using an ensemble of protein states guided by different types of experimental restraints. Oligomers that form from these precursors may retain the structure of the monomer, convert to a different structure, or form new structures not accessible to their precursors. All eventually form the cross-β fold of amyloid which, whilst containing a canonical parallel in-register β-strand structure can adopt a variety of different structures (127 different amyloid structures have been collated in the amyloid atlas; Sawaya et al., 2021).

Despite this extraordinary progress, and the stunning advances in structural methods such as cryo-EM, cryo-ET and solid state NMR over the last few years (Bäuerlein and Baumeister, 2021; Reif et al., 2021; Saibil, 2022), generating high resolution structures of aggregation intermediates remains enormously challenging, and the secrets of protein misfolding remain unveiled. Understanding the early events in protein misfolding that result in large-scale self-assembly into the highly ordered cross-β fibrous assemblies of amyloid is challenging from the physical chemistry view point (Cawood et al., 2021). Intrinsic protein dynamics play a crucial role in the early stages of the misfolding reaction. These can be manifested in the form of intrinsically disordered proteins (IDPs) (Bondos et al., 2021; Uversky, 2021) that exchange between an array of different conformations, but also partially folded amyloid precursors that retain a dynamic 3D structure, which can loosely self-assemble to generate a pool of low-order oligomers (Figure 1). Thus, the first main challenge in understanding the principles of protein misfolding is the ability to generate ensembles that capture the dynamics of aggregation precursors that can span the ns to hour timescale. However, the majority of these states may be innocuous in terms of amyloid formation, since they will not possess the physico-chemical properties required to enter the aggregation landscape and will remain monomeric or, if forming inter-molecular interactions in oligomers, will disassemble back to monomers with which they are in dynamic equilibrium (Dear et al., 2020; Michaels et al., 2020; Cawood et al., 2021). This represents the second main challenge: how do we identify specific sub-ensembles within large pools of interconverting species that show increased propensity to aggregate and/or assemble into amyloid?

Here, we review current computational and experimental methods that can be used to describe the solution properties of highly dynamic proteins, with emphasis on how the kinetics of their formation can influence the structural interpretation of experimental observables. We then discuss how clustering of these ensembles may be performed using machine learning methods in order to identify aggregation-prone vs. aggregation-resilient states. Finally, we show how these methods/concepts can be used to describe the misfolding of two example systems: a protein that aggregates from an IDP state (αSyn) or from a dynamic, yet topologically well-defined species (β_2_m).

## Misfolding Proteins Across the Flexibility Scale

Proteins with an enhanced propensity to aggregate into amyloid can be (1) disordered (IDPs) or contain intrinsically disordered regions (IDRs), (2) structured, but unstable thermodynamically or kinetically, or (3) combinations of these traits. Examples include variants of immunoglobulin light chain associated with light chain amyloidosis (thermodynamically unstable) (Morgan et al., 2021), β_2_m (both kinetically and thermodynamically unstable; Eichner and Radford, 2011), amyloid-β (Aβ), α-synuclein and islet associated polypeptide (IAPP) that are IDPs (Chiti and Dobson, 2017) and prion protein (Singh and Udgaonkar, 2015) or poly-glutamine-containing proteins such as ataxin 3 and huntingtin (contain both structured and disordered regions) (Lieberman et al., 2019).

For IDPs, disorder serves as a means to explore a vast conformational landscape in their monomeric form that may, or may not, be related to their function and/or propensity to aggregate. Thermodynamically, for disordered proteins to aggregate into amyloid, the gain in enthalpy from the formation of the repetitive cross-β interactions (main-chain hydrogen bonding and interactions between stacked side-chains) of the ubiquitous amyloid fold compensates for the entropy loss arising from the ordering of a disordered/unstructured polypeptide chain. Disorder that leads to misfolding can also be generated by other mechanisms, including proteolytic cleavage of larger precursors that may be otherwise folded/aggregation-resilient (serum amyloid A, antibody light chains, transthyretin, Aβ, and others) (Adams et al., 2019; Lewkowicz and Gursky, 2022; Lichtenthaler et al., 2022) or even aggregation of the nascent polypeptide chain as it exists the ribosome (Willmund et al., 2013; Deuerling et al., 2019; Cassaignau et al., 2020).

For protein precursors that are initially folded (e.g., β_2_m, light chains, transthyretin; Iadanza et al., 2018), local protein motions which lead to exposure of hydrophobic/aggregation-prone regions (APRs) (Beerten et al., 2012; Houben et al., 2022) that are normally buried in the native structure, have been suggested as the drivers of self-assembly. For misfolding-prone proteins that contain long disordered regions (IDRs) dispersed within, or at the termini, of folded domains (e.g., prions and polyQ-containing proteins), the initiating stages of aggregation may be dominated by the IDR, by interactions involving the folded domain, or both (Scarff et al., 2013; Singh and Udgaonkar, 2015; Sicorello et al., 2018, 2021; Lieberman et al., 2019). And, while it is now straightforward to predict the presence of APRs in protein sequences (Tsolis et al., 2013), these regions cannot be solely responsible for driving the initial stages of aggregation, since it is well-known that regions that flank these sequences can play a pivotal role in controlling assembly (Ulamec et al., 2020). The small oligomeric species that self-assemble from aggregation-prone monomers can have “memory” of the structural properties of their corresponding precursors, thus creating pools of native, partially folded or unfolded oligomers (Cawood et al., 2021). Alternatively, self-assembly may generate new structures not accessible/populated in their monomeric precursors (Figure 1). Initially formed small oligomers can continue to grow in size, without further conformational change to generate larger amorphous aggregates, or they can undergo a transition to a cross-β structure which is followed by elongation processes that result in the formation of the large fibrillar aggregates classic of amyloid (Xue et al., 2008; Knowles et al., 2009).

In summary, therefore, even by focusing on the earliest stages of misfolding and aggregation a complicated picture emerges that involves numerous, structurally distinct precursors that lead to aggregate formation via a range of kinetic mechanisms. Nonetheless, the vast diversity of protein structures of unrelated sequence and function that can form fibrillar aggregates suggests the presence of common, fundamental underlying mechanisms that are yet to be discovered and understood.

## Experimental Methods Used to Guide the Generation of Protein Ensembles

The conformational heterogeneity of IDPs and proteins that contain a significant portion of IDRs precludes the conventional investigation of these species using methods able to determine high resolution structures, such as cryo-EM and X-ray crystallography (Thomasen and Lindorff-Larsen, 2022). For amyloid precursors that are initially folded, even though the native monomeric state may be populated to an extent that allows its characterization by structural approaches, these methods cannot capture the rarely populated, partially folded species that can be crucial for aggregation (Radford et al., 1992; Dhulesia et al., 2010; Buell et al., 2011; Karamanos et al., 2016), or the loosely associated oligomeric species that form early during assembly (Laganowsky et al., 2012; Karamanos et al., 2014, 2019; Fusco et al., 2017). Methods able to capture both local and global properties of the polypeptide chain, and to detect rare and transiently populated species, are needed in order to describe the conformational properties of these dynamic protein states. Since the equilibria that lead to the formation of these lowly populated species are uniquely sensitive to factors such as pH and salt concentration, and hence the rate of amyloid aggregation is also highly dependent on the solution conditions (Buell et al., 2014), experimental restraints should ideally be collected in solution. Experiments need to be carefully planned so that these early species are resident for long enough to enable their detection and characterization, holding off the inevitable downhill thermodynamic cascade to the amyloid fold (Karamanos et al., 2015). If such conditions can be found, a range of powerful solution techniques can be used to yield restraints used in ensemble calculations (Cawood et al., 2021). These include small angle X-ray scattering (SAXS)/NMR (Mertens and Svergun, 2017) (generating R_h_), hydrogen exchange (HX) monitored by NMR or mass spectrometry (MS) (Radou et al., 2014; Wan et al., 2020) (yielding information on solvent accessibility of the main-chain/hydrogen bond stability); single molecule fluorescence energy transfer (smFRET) or fluorescence correlation spectroscopy (FCS) (Naudi-Fabra et al., 2021) (interatomic distances and distance distributions), and chemical cross-linking (Faull et al., 2019) (inter-residue contacts). Alternatively, in favorable cases, restraints collected in the gas phase by electrospray ionization mass spectrometry (ESI-MS) (Politis et al., 2014; Rajabi et al., 2015; Österlund et al., 2019) (ion mobility, mass distribution) or in the frozen state using electron paramagnetic resonance (EPR) (Jeschke, 2018; Kapsalis et al., 2019) (distance distributions) can provide additional information, as long as the ionization/freezing process can be ensured not to change the conformational equilibrium.

While each of these methods alone cannot deal with the vast heterogeneity of protein ensembles in terms of the array of different protein conformations and oligomeric states present, when applied together the properties of these complex systems can begin to be revealed (Gomes et al., 2020; Naudi-Fabra et al., 2021). A prerequisite for an experimental restraint to be used in the generation of a conformational ensemble is that its value must be able to be directly back-calculated from the atomic coordinates of the species present. This is not always a simple task, as it generally requires a robust theoretical model that can take into account the extreme averaging that takes place in highly dynamic proteins. In the next paragraphs we give a brief overview of some of the techniques that have been used to generate ensemble representations of misfolding proteins. For a more technical description of how these methods work we refer the reader to a number of excellent reviews (Roy et al., 2008; Clore and Iwahara, 2009; Jeschke, 2012; Politis et al., 2014; Chiliveri et al., 2021).

A technique that naturally ticks all the boxes for analysis of dynamic protein ensembles in solution is NMR spectroscopy. NMR is the go-to method when disordered proteins or proteins with IDRs are involved (Meier et al., 2008; Jensen et al., 2013; Arai et al., 2015; Salvi et al., 2016; Dyson and Wright, 2021). Its unique ability to provide residue-specific information in solution (using ^1^H, ^13^C, and/or ^15^N labeled proteins) is one of the main advantages that make NMR stand out from other biophysical techniques (Alderson and Kay, 2021). Solution NMR can be used to provide numerous experimental observables that report on local (chemical shifts, short range nuclear Overhauser effects (NOEs), 3-bond J couplings) or global [residual dipolar couplings (RDCs), paramagnetic relaxation enhancements (PREs)] properties of a proteins’ structure. Importantly, NMR spins are sensitive to the overall tumbling of the molecule and also to local motions, and thus sophisticated NMR relaxation methods can be used in order to study protein motion directly (Lipari and Szabo, 1982a,b). Of the many NMR methods available, the ones that report on global, slower timescale motions (such as RDCs and PREs) are perhaps more useful in order to capture the large-scale dynamics of misfolding proteins and thus we will focus our discussion on those (see Table 1 for a more comprehensive list). The well-known molecular weight limitation of NMR which make the study proteins > 30 kDa in size difficult, unless specific labeling (e.g., ^13^C methyl) is used (Tugarinov and Kay, 2004), is not prohibitive for IDPs (even if these consist of more than 300 residues; Mamigonian et al., 2022), since local disorder causes long transverse relaxation (T_2_) times and therefore NMR signals do not decay rapidly. For natively folded proteins that interconvert with misfolded monomeric or oligomeric states, the properties of the misfolded/aggregated state can also be investigated by adjusting the solution conditions such that misfolded states represent a small fraction of the molecules in solution, allowing powerful NMR methods to characterize excited, rarely populated (<5%) protein states (Anthis and Clore, 2015). When these experiments are performed and data successfully obtained, calculating NMR observables from structure can be straightforward. This is certainly the case for distance-based measurements (NOEs) which are often calculated as an *r*^–6^ weighted average of the interatomic distances *r*. However, other NMR observables, such as chemical shifts, do not have analytical expressions to describe their relationship with atomic coordinates, and empirical models are often used (Shen et al., 2008; Robustelli et al., 2010, 2012). It is important to keep in mind that the timescales of exchange between the various protein states, which could represent transiently folded regions of IDPs or IDRs, or monomer-oligomer equilibria, also affect the NMR observables, and how the kinetics of exchange affect a particular NMR parameter has to be taken into account for a quantitative interpretation of the data (Salvi et al., 2016) (see following section).

**TABLE 1:** NMR methods and their usage in ensemble calculations.

NMR method	Advantages/Disadvantages in ensemble calculations	Usage
RDCs	Long range reporters of structure and (in some cases) dynamics	Frequent
PREs	Long range distance restraints	Frequent
Chemical shifts	Easy to obtain, can be related to local protein structure using empirical relationships	Frequent
Order parameters/spectral densities	Local, ps—ns motions	Frequent
Chemical exchange methods	Probes of ms or slower motions, require extensive sampling	Indirect as chemical shift restraints
Hydrogen exchange	Good models to predict hydrogen exchange rates still lacking	Rare
Diffusion NMR	Difficult to extract hydrodynamic parameters of individual species without prior knowledge of species populations	Rare

The atom-specific information obtained from NMR studies is even more powerful if it can be combined with other techniques that provide complementary information such as smFRET or SAXS (Krzeminski et al., 2013; Lincoff et al., 2020; Naudi-Fabra et al., 2021). smFRET measures the proximity of individual pairs of fluorescence dyes over time (in TIRF mode) or population (in confocal mode) and thus can inform on conformations of individual molecules and the kinetics of their interconversion in a quantitative manner (Roy et al., 2008; Schuler and Eaton, 2008). In smFRET studies, care must be taken to ensure that the fluorescent dyes do not alter the proteins’ properties which is of key concern for IDPs/IDRs (Borgia et al., 2016). Small angle X-ray scattering (SAXS), on the other hand, is a dye-free ensemble technique that reports on the overall shape of the protein under investigation and can be used to derive the overall compactness of the ensemble by weighting various conformations present in solution (Różycki et al., 2011; Ahmed et al., 2021). Both techniques have been used extensively to generate ensemble representations of IDPs or multidomain proteins which contain a significant portion of IDRs (Bernadó et al., 2005b; Merchant et al., 2007; Holmstrom et al., 2018). In terms of aggregating proteins, integrative studies have been performed in order to describe ensembles of ataxin (Sicorello et al., 2021), α-syn (Schwalbe et al., 2014; Chen et al., 2021), amyloid β (Sgourakis et al., 2007) and tau (Chen et al., 2019; Stelzl et al., 2022) among others (Strodel, 2021).

A technique that is powerful, but perhaps under-utilized, when it comes to dynamic proteins is ESI-MS. Bottom up ESI-MS experiments can provide restraints captured in solution and analyzed subsequently using liquid chromatography MS (LC-MS) (such as cross-linking or HX studies) (Belsom and Rappsilber, 2021) or native ESI-MS that is performed on intact molecules in the gas phase (Beveridge and Calabrese, 2021). Ion mobility MS that reports on the collision cross section (CCS) of a protein can separate species based on mass (monomer, dimer etc.), but can also resolve species of the same mass, but different CCS (e.g., compact vs. expanded versions of isobaric species) (Beveridge et al., 2019; Moons et al., 2020). The ability of native ESI-MS to detect small populations of protein conformers and separate them based on size (resolution of a few Da) and shape (CCS) has been powerful in the investigation of folding/misfolding and aggregation pathways (Benesch et al., 2006; Smith et al., 2006, 2007; Woods et al., 2013; Young et al., 2014; Britt et al., 2021) and in the assembly of dynamic chaperone assemblies (Young et al., 2018). Theoretical models that allow the calculation of MS-derived restraints such as CCS are perhaps lacking, although significant progress in this area has been made recently (Kulesza et al., 2018). Concerning IDPs or IDRs, it is important to ensure that the compaction or extension of the polypeptide chain observed is not the result of the electrospray ionization/desolvation process itself (Vahidi et al., 2013; Borysik et al., 2015; Devine et al., 2017).

To avoid ionization issues, experiments that capture protein motions can be performed in solution and subsequently analyzed by MS methods. Zero-length cross-linkers (such as EDC (1-ethyl-3-(3-dimethylaminopropyl) carbodiimide hydrochloride) and DMTMM (4-(4,6-dimethoxy-1,3,5-triazin-2-yl)-4-methylmorpholinium chloride)) allow adjacent carboxyl and amine-carboxyl sidechain to be covalently linked and identified using proteolysis and tandem MS (LC-MSMS). An array of cross-linkers with different chemistry (free-radical, maleimide, NHS ester, and others) and cross-linker length, can provide additional information on sidechain-sidechain distance, albeit averaged over the timescale of the cross-linking experiment (Sinz, 2018). Using lasers or LEDs the timescale needed for photo-crosslinking can be reduced from tens of minutes to less than seconds (Russmann et al., 1998), providing a clearer snap-shot of the interactions by reducing averaging (Horne et al., 2018). These experiments capture the dynamic nature of intra/inter-molecular contacts and combined with computational analysis can visualize these species (O’Reilly and Rappsilber, 2018; Zamel et al., 2021). Even though in some cases crosslinking restraints have been treated as NMR-derived distances, care has to be taken when dealing with ensembles of structures, since the nature of the two distances is fundamentally different. Once an irreversible crosslink has formed, the two atoms are not available for any further additional reactions, whereas in an NOE experiment one atom may give rise to multiple distance restraints.

Differential hydrogen—deuterium exchange that measures the solvent accessibility/hydrogen bond stability of the protein under investigation is another technique that combined with ESI-MS analysis can be used to investigate large/dynamic states at the peptide/single residue level (by rapid quenching, proteolysis and LC-MSMS analysis) (Faull et al., 2019; Calabrese et al., 2020; Wang et al., 2022). Recent innovations have also increased the time resolution of HX-MS to ms (Hu et al., 2013; Seetaloo et al., 2022). These data can be converted to protection factors and can be used for ensemble generation (Wan et al., 2020). Using sophisticated pulse schemes, hydrogen exchange with solvent can be followed by NMR that allows ultra-fast, sub-ms rates to be measured without the need of dedicated HDX hardware (Skrynnikov and Ernst, 1999; Kateb et al., 2007; Segawa et al., 2008; Dass et al., 2021). One drawback of hydrogen exchange methods that limits their application toward ensemble generation is that accurate models that describe the crucial role of electrostatics to the measured exchange rates are lacking (Table 1).

We note that although the techniques mentioned in the previous paragraphs are excellent in capturing the soluble species formed in the early stages of protein aggregation, the reduced solubility of aggregates formed later in assembly may limit the repertoire of solution techniques available to characterize them. Such states are perhaps best captured by techniques such as cryo-EM (Bäuerlein and Baumeister, 2021; Saibil, 2022), solid-state NMR (Reif et al., 2021) and/or atomic force microscopy (AFM) (Aubrey et al., 2020). Despite recent advances, sample heterogeneity still poses significant challenges in the characterization of partially soluble states (Cawood et al., 2021). Overall, it is clear that many experimental techniques must be used to generate complementary restraints that together have the potential to visualize the dynamics that are in play.

## Ensemble Averaging of Experimental Restraints

**FIGURE 2:**
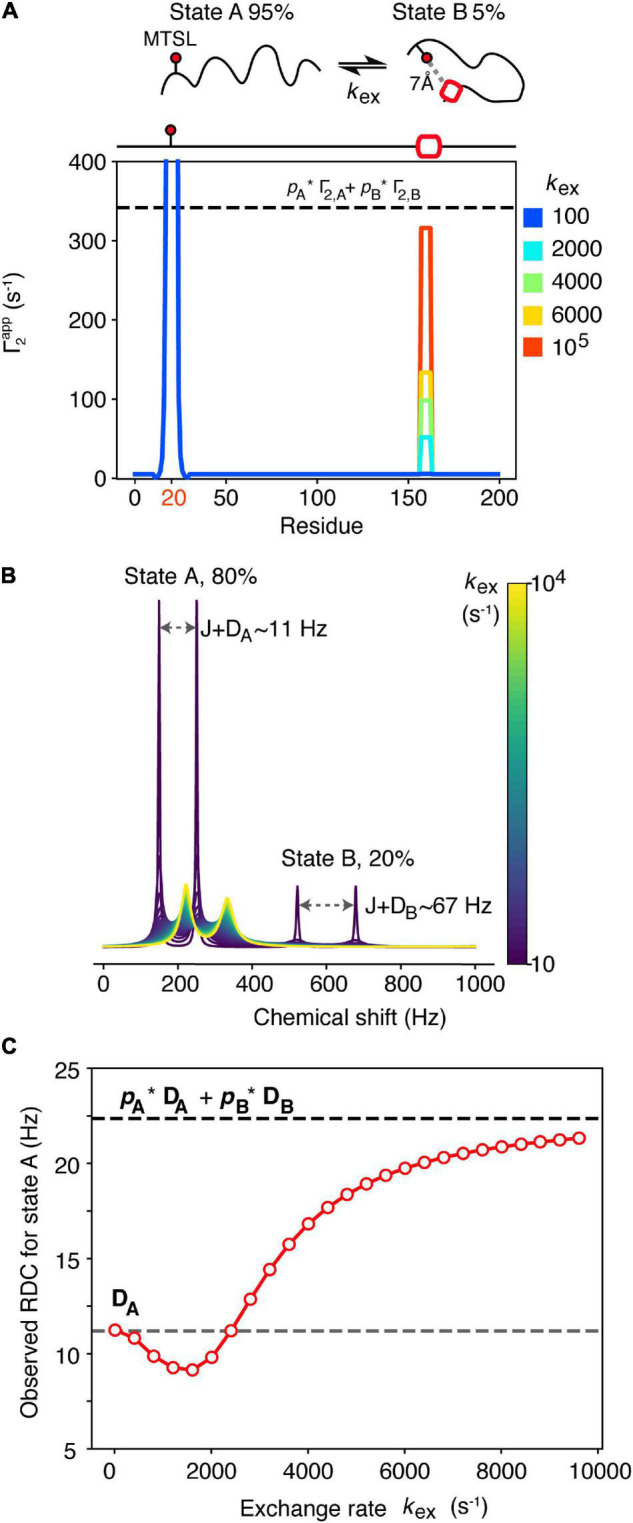
Dependence of NMR observables often used to drive ensemble generation on the kinetics of chemical exchange. **(A)** A spin-labeled IDP undergoes intramolecular exchange between an expanded state A (*p*_A_ = 95%) and a more compact state B (*p*_B_ = 5%) that involves transient helix formation (red box). The 7Å distance between the spin label (placed on residue 20 of this hypothetical 200 residue protein) and the helix in residues 157–163 in state B gives rise to a PRE rate for that state, Γ_2,B_ = 6,750 s^–1^, while the PRE rate for state A where these residues are > 15 Å apart is low (Γ_2,A_ = 5 s^–1^). Only when exchange is fast on the PRE timescale (*k*_ex_ ≫ Γ_2,B_) does the observed PRE rate approximate the population-weighted average (dashed line). **(B)** Simulated 1D NMR spectra of a 2-spin coupled (coupling constant *J* = 90 Hz) system that undergoes 2-site exchange. State A is highly populated *p*_A_ = 80% and gives rise to an RDC D_A_ = 11.2 Hz, while the RDC of state B, D_B_ is 67 Hz. The chemical shift of state A was set to ω_A_ = 200 Hz, giving rise to a doublet, separated by *J*+D_A_. For state B, ω_B_ = 600 Hz. Simulated spectra at different exchange rates (*k*_ex_, colored bar) were generated using 5,000 points, apodised and Fourier transformed. O wing to the small value of *p*_B_ = 20%, the doublet corresponding to state B (separated by *J*+D_B_) is only visible in the first spectrum. The state A doublet moves toward the average chemical shift position with increasing *k*_ex_. Peak positions and linewidths were extracted using a Gaussian fitting procedure. The RDC of state A was measured as the difference in frequency of the state A doublet after *J* was subtracted, and is plotted as a function of *k*_ex_ in **(C)** (red dots). Under slow exchange the observed RDC equals D_A_ (gray dashed line) while under fast exchange it approximates the population weighted average (black dashed line). The reduction in the observed RDC values observed in the intermediate exchange regime arises due to artifacts in determining peak positions when linewidths are larger than J+D.

For most of the experimental techniques mentioned above, theoretical frameworks that allow the back-calculation of the experimental restraints from the molecular structure exist. However, when dealing with highly dynamic proteins such as those involved in protein misfolding and aggregation, these restraints need to be averaged appropriately in order to generate an accurate representation of the solution properties of the entire ensemble. It is often the case that the different protein states within the ensemble are assumed to be in fast exchange between each other. This essentially means that the exchange between these species is faster than observation of the experimental variable, and thus the experimental restraints correspond to the population-weighted average between all the conformers. Fast exchange is supported by the poor chemical shift dispersion of IDPs (IDRs) in NMR studies, and is usually a safe approximation for these types of proteins, but it may not always be the case. Protein self-oligomerization that occurs in the early stages of aggregation, or even the formation of local secondary structural elements in IDPs, can occur on slower timescales. In the case of NMR observables, the kinetics of the conformation exchange can significantly affect the measured values (Iwahara and Clore, 2006; Cavalli et al., 2013; Janowska and Baum, 2016). Figure 2A shows how PREs are affected by the kinetics of exchange between an extended (state A) (95% populated) and a rarely populated (5%) compact state B in which a hypothetical C-terminal helix is interacting with the N-terminal segment of a protein. In the compact state (state B) the distance (*r*) between the spin label [usually S-(1-oxyl-2,2,5,5-tetramethyl-2,5-dihydro-1H-pyrrol-3-yl) methyl methanesulfonothioate (MTSL)] and the helix is 7 Å, giving rise to a high PRE value (or Γ_2,B_) rate (6,750 s^–1^), while the PRE rate for state A, in which the spin label and N-terminus is > 15 Å away, is low (Γ_2,A_ = 5 s^–1^). In the fast exchange limit, wherein the rate of exchange *k*_ex_ ≫ Γ_2,B_ the observed PRE rate approximates the population weighted average of Γ_2,A_ and Γ_2,B_ (Clore and Iwahara, 2009; dashed line in Figure 2A). However, if *k*_ex_∼Γ_2,B_ or *k*_ex_ < Γ_2,B_ the observed PRE rate is much smaller than the population weighted average (Figure 2A). In this hypothetical case the rate of N-C association could be determined, in principle, from the rate of helix formation (assuming that helix formation can only occur when the termini come into close contact), but of course, in reality helix formation could be slower than the rate of binding. Clearly, for four out of the five curves in Figure 2A the fast exchange assumption would lead to overestimation of r and the generation of a more expanded ensemble that could fit the experimental data equally well.

RDCs can also provide useful information about protein structure and are powerful when using NMR to calculate structures and dynamics of proteins (Chiliveri et al., 2021). For dynamic systems, RDCs are normally averaged following two assumptions: (1) That all possible conformations can be sampled during the measurement time and (2) that interconversion between states is slower than the event that leads to re-orientation of the molecule in the alignment medium [related to the correlation time (τ_c_) of the molecule] (Meier et al., 2008). If both assumptions are satisfied, transformation from the time average to the ensemble average is straightforward, and the observed RDC will be equal to the average over all molecular conformations. In general, assumption 2 is normally a safe assumption, as molecular reorientation should be very fast and comparable to the molecular tumbling time (on the ns timescale), unless association of the protein with the alignment medium takes place. For highly dynamic IDPs, assumption 1 should also be satisfied, but this might not be the case if transient interactions are formed that result in conformational exchange on a slower timescale (Figures 2B,C). Imagine a scenario in which an IDP (state A) exchanges with a transiently folded state (state B) that may be related to misfolding. Alignment of state A may be weak (as it is normally the case for IDPs), giving rise to an RDC for that state, D_A_ = 11.2 Hz, while the folded state B gives rise to D_B_ = 67 Hz. As observed in Figure 2A for PREs, the measured RDC for both states depends on the exchange rate between them. For simplicity we will discuss only state A, as state B is populated only to 20% in this example, and may not be directly observable (Figure 2B). In the slow exchange limit on the chemical shift timescale (*k*_ex_ < 100 s^–1^) the observed RDC for state A equals D_A_, while when exchange approaches the fast exchange regime (*k*_ex_ > 8,000 s^–1^) the observed RDC approximates the population-weighted average of the two states, as expected (Lorieau et al., 2012; Figure 2C). However, it is evident from Figure 2C that in the intermediate exchange regime (100 < *k*_ex_ < 8,000 s^–1^) the observed RDC shows a complex behavior that, if not correctly taken into account, may lead to erroneous conclusions about presence/absence of local secondary structure, for instance, in a dynamically interconverting ensemble of states.

In conclusion, treating NMR-derived restraints as populated-weighted averages over all ensemble members is able to capture the time averaging that happens in solution when exchange between the various states is fast. This has led to some elegant examples including the generation of ensembles of misfolding IDPs able to quantitatively describe the experimental restraints (Iwahara et al., 2004; Bernadó et al., 2005a; Dedmon et al., 2005; Huang and Grzesiek, 2010; Salmon et al., 2012; Janowska et al., 2015; Salvi et al., 2016; Karamanos et al., 2019; Naudi-Fabra et al., 2021; Sicorello et al., 2021; Mamigonian et al., 2022). However, when/if motions on slower timescales occur, these have to be taken into account in order to avoid data misinterpretation.

## Converting Experimental Restraints Into Ensembles of Structures

Different computational approaches have been developed that enable measured experimental restraints to be converted into structural ensembles. The two main approaches involve (1) biasing molecular dynamics (MD) simulations by the addition of energy terms that minimize the difference between the observed and calculated restraints (Jaynes, 1957; Roux and Weare, 2013), or (2) reweighting ensembles that have been initially generated with no experimental information (Różycki et al., 2011; Cavalli et al., 2013). In both cases overfitting is avoided using maximum entropy or Bayesian techniques. Approach 1 requires that the theoretical models used to calculate the experimental observables from structural models are also differentiable, which sometimes is not straightforward, especially for some of the MS-derived restraints (such as CCS). Approach 2, on the other hand, assumes that all relevant protein states are already present in the initial ensemble and may not be appropriate in cases where conformational sampling is not efficient. A detailed description of these computational protocols is beyond the scope of this review and we refer the reader to some excellent recent reviews on the topic (Hummer and Köfinger, 2015; Bonomi et al., 2017; Bottaro and Lindorff-Larsen, 2018; Pietrek et al., 2020; Thomasen and Lindorff-Larsen, 2022). We note that the computer-generated ensembles are only a true reflection of the experimental data that were used for their generation. Parameters such as the number of ensemble members or even their weights can vary depending on the nature and quantity of the experimental input. Hence, the more complex and broad the number of conformers, the greater the number of experimental data of different type is needed to best define the ensemble. Thus, a plethora of different, unrelated experimental methods are needed in order to obtain an unbiased representation of the dynamics that take place in solution. We note that the recent developments in deep learning algorithms able to accurately predict the structure of folded proteins from their amino-acid sequence opens the window for a future extension of these methods to capture hidden structural motifs/propensities in IDPs. In order for this to happen, a large, high quality dataset of experimentally determined ensembles (using the methods described here) is necessary in order to train accurate deep learning networks. Although this is not available at the moment, the fast progress in the field of protein chemistry holds for an exciting future in this research area (Serpell et al., 2021).

## Extracting Information About Misfolding/Aggregation Sub-Ensembles Using Machine Learning. An Example From α-Synuclein

Of the vast number of species contained in an ensemble of monomeric aggregation-prone IDPs, and oligomeric ensembles of folded/unfolded precursors, only a tiny minority of conformers may possess the properties required for further aggregation. Of all possible conformers, only specific sub-ensembles will be able to transition into the aggregation landscape and eventually push the equilibrium toward fibrillar species that lie at a thermodynamic energy sink (Figure 1). How can one then search for, or tease-out, aggregation-relevant members of the ensemble from their aggregation-resilient counterparts? The answer to this question is not obvious currently, but its solution would represent a key step forward in understanding how, and why, proteins aggregate. Building on recent advances in the field of machine learning, we discuss below how such techniques can be used to generate new insights into aggregation-relevant conformers buried within a myriad of alternative species unrelated to an aggregation pathway into amyloid.

The problem of sub-clustering of structures based on common properties is not a new one, and techniques such as principal component analysis (PCA) are elegant ways to generate sub-clusters based on overall similarities in one or more structural properties (Papaleo et al., 2009). In many ways, ensemble sub-clustering resembles problems that are ideal for unsupervised machine learning methods, that are typically described as an unbiased method to identify patterns in “unlabeled” data (unlabeled here refers to the fact that each structure is not tagged *a priori* with a label that includes it to cluster X). In its simplest form unsupervised clustering can be performed by Gaussian mixture models (GMM) that, given a number of normal distributions, will try and determine to which distribution each point belongs. The number of normal distributions the model has access to is usually not known and may affect the clustering results. Thus, these models are often combined with Bayesian approaches to keep the number of distributions to a minimum (Roberts et al., 1998).

**FIGURE 3:**
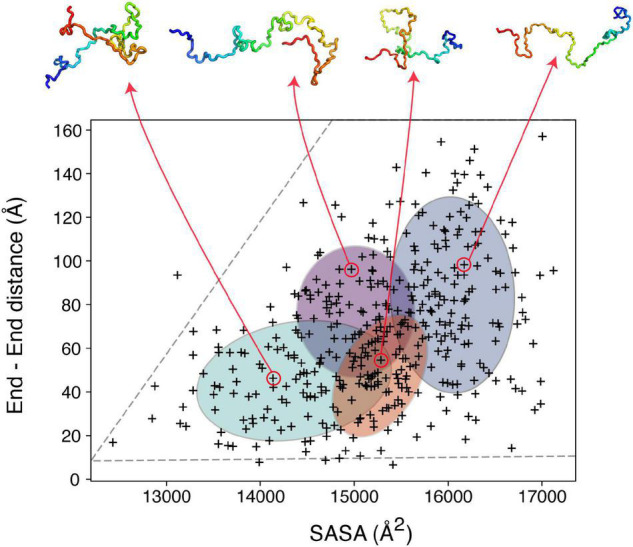
Clustering IDP ensembles using machine learning. A Bayesian Gaussian mixture model to classify an αSyn ensemble that consists of 400 structures based on their end-to-end distance and solvent accessible surface area (SASA). The four ellipses correspond to the four clusters identified with four structures shown as examples above. The ensemble used for this analysis (PED00024) was generated by Allison et al. (2009), using MD simulations driven by PRE restraints collected in 10 mM sodium phosphate pH 7.4, 100 mM NaCl, 10°C.

**FIGURE 4:**
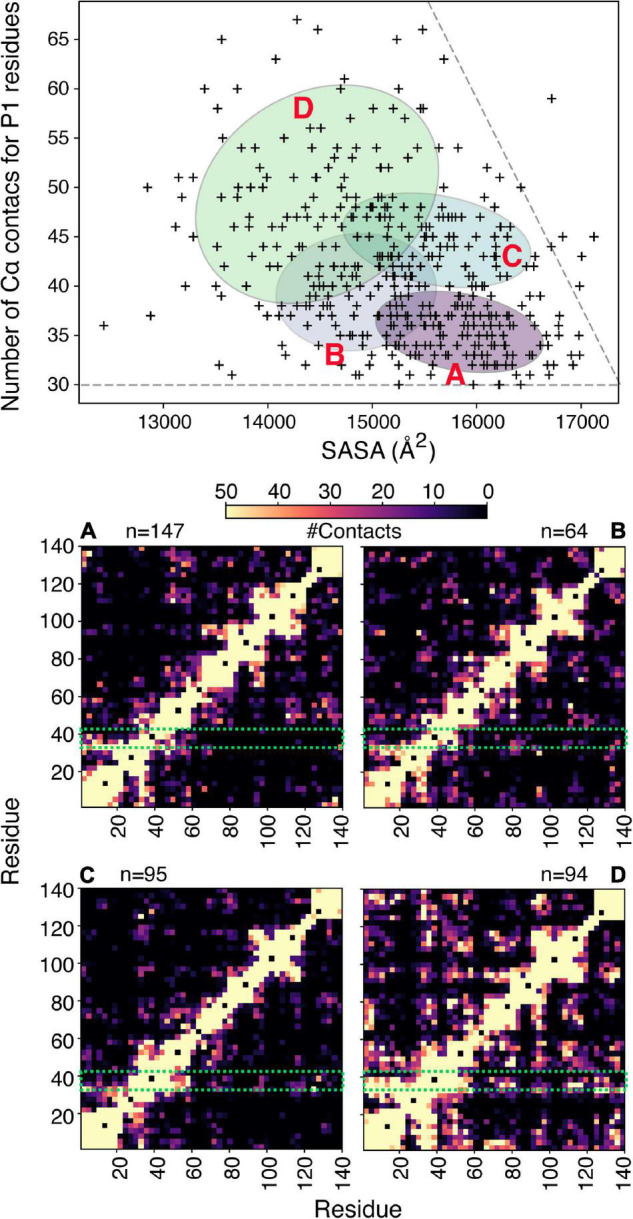
Clustering of αSyn conformers based on aggregation-prone regions. **(A)** Bayesian Gaussian mixture model to classify an αSyn ensemble (Allison et al., 2009) that consists of 400 structures shown in Figure 3, based on the number of Cα contacts made by residues in P1 and the solvent accessible surface area. For clustering a contact is defined if two Cα atoms are within 8 Å. The four ellipses correspond to the four clusters identified and are labeled **(A–D)**. The corresponding contact maps are shown below. For the contact maps the definition of contacts is more generous and includes all atoms of two given residues. The P1 region is highlighted in a green box and n denotes the number of structures in each cluster.

To illustrate the power of clustering methods based on machine learning we use here an ensemble of αSyn structures that was generated using molecular dynamics simulations guided by 595 NMR PRE-derived intramolecular distances (Allison et al., 2009). Figure 3 shows the performance of a simple GMM in clustering of αSyn structures based on their end-to-end distance and surface accessible surface area (SASA). Four partially overlapping clusters are evident, although there is definitely room for improvement. Instead of performing clustering analysis using global features as shown in Figure 3, we can extend these ideas to include local features. Due to the complex nature of the problem, in many cases information about which residues/regions of the protein are important/irrelevant for misfolding/aggregation is sparse (Aguirre et al., 2022; Seetaloo et al., 2022). Perhaps the most informative results come from mutational studies that assess the effect of mutations on misfolding/aggregation rate in a rigorous way. For instance, we have recently shown that a 7-residue segment (residues 36–42), termed P1, in the N-terminal region of αSyn acts as a “master regulator” of aggregation (Doherty et al., 2020). Deletion or substation of the seven residues in P1 prevents aggregation of αSyn at neutral pH *in vitro* (up to the experimental time of 100 h) and also prevents amyloid formation and proteotoxicity in *C. elegans* (Doherty et al., 2020). NMR PRE experiments showed that residues in P1 make extensive intramolecular contact with the NAC region that this region flanks, as well as the acidic C-terminal region of the protein (Doherty et al., 2020). Yet, how these contacts alter or refine the structural ensemble, and how these changes “turn on” aggregation of the protein remains obscure at a molecular level. Do residues in P1 show specific intra-molecular interaction hidden within the broad ensemble of conformers shown in Figure 3, and do these interactions result in compaction/other alterations of the chain? To answer these questions, we trained another simple Bayesian GMM to cluster the αSyn ensemble based on the number of contacts made by residues in P1 and the SASA. The four clusters shown in Figure 4 range between expanded conformations with very few P1 contacts (cluster A) to more compact states with more contacts made by residues in P1. All four clusters show differences in their contact maps, with clusters A and D being most different. Even though this analysis is used here only for illustration purposes, it highlights the type of information that can be gained. For instance, interactions between residues in the important NAC region (residues 61–95) are only present in cluster D when P1 is also involved in numerous contacts with the NAC and C-terminal regions, while in cluster A NAC seems to be shielded by the C-terminus (Figure 4). Although the use of machine learning described here is solely to unpick already available dynamic ensemble, other uses of these powerful methods can be envisaged, such as in molecular dynamics simulations used to generate the initial ensemble (Noé et al., 2020). In general, we expect that these types of analyses, extended to deep convolutional neural networks, will reveal hidden patterns and propensities for IDPs, much like they were able to revolutionize structure prediction for folded proteins.

## Ensembles of Transient Oligomeric Species Formed by Folded Precursors. An Example From β_2_M

**FIGURE 5:**
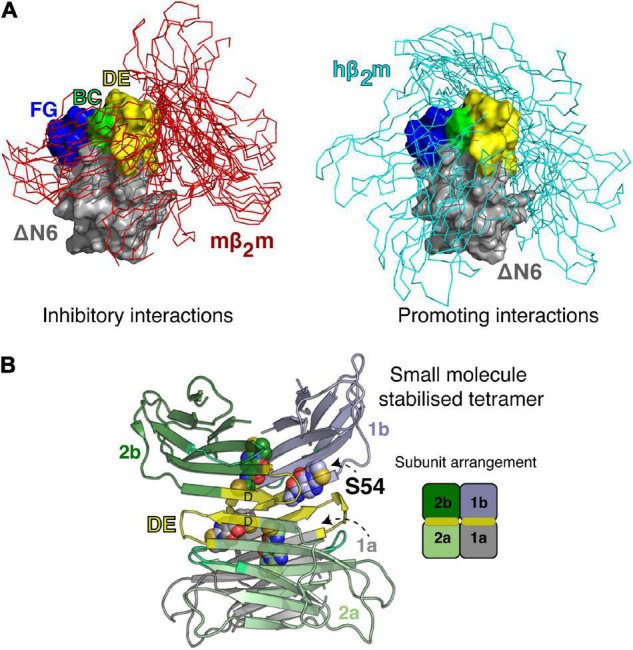
Transient and stable forms of β_2_m oligomers. **(A)** Ensembles showing the dynamic nature of the ΔN 6-mβ_2_m (left) and ΔN 6-hβ_2_m (right) interactions (Karamanos et al., 2014). ΔN 6 is shown as a Cα trace with the BC (green), DE (yellow) and FG (blue) loops highlighted (space fill). Note that the BC loop contains the trans Pro32. Hβ_2_m and mβ_2_m are shown in a surface representation (gray) bound to ΔN 6. E nsembles of 100 complexes (aligned on ΔN 6) are shown. **(B)** A small molecule stabilized tetramer of ΔN 6 (7AFV) (Cawood et al., 2020). ΔN 6 subunits are shown as cartoons and the four copies of the covalent small molecule (S54; Cawood et al., 2020) are highlighted as spheres. The DE loop that is involved in one of the tetramer interfaces is shown in yellow and a schematic of the subunit arrangement in the tetramer is shown on the right.

Many of the ideas described above for defining and sub-classifying the monomeric ensembles of IDPs, are equally well applicable to address the challenges with understanding early oligomeric species formed by specific assembly of partially folded protein conformers, as such species are also often highly heterogeneous, dynamically interconverting and short-lived. Structural information for several of oligomeric intermediates of amyloid assembly is available, in cases where these species have been trapped/enriched by specifically designed chemical tools or caught by NMR, MS or single molecule methods (Cawood et al., 2021). However, these examples are far less numerous than those of IDPs. This reflects the difficulty in finding conditions wherein stable populations of oligomeric species are present, without further polymerization into amyloid fibrils. One such system with favorable properties for biophysical analysis is wild-type human β_2_m (hβ_2_m), the culprit protein of dialysis related amyloidosis (Gejyo et al., 1985). hβ_2_m is highly resistant to aggregation *in vitro*, and its polymerization *in vivo* is thought to be initiated by partial unfolding on the surface of collagen filaments (Relini et al., 2006; Hoop et al., 2020). The propensity of hβ_2_m to aggregate into amyloid is also enhanced dramatically by proteolytic cleavage of six amino acids from its N-terminus, which generates a highly aggregation-prone and partially folded variant, ΔN 6 (Esposito et al., 2000; Eichner et al., 2011; Karamanos et al., 2014). While ΔN 6 retains a native-like immunoglobulin fold, the protein is far from native; it is dynamic and weakly protected from hydrogen exchange, contains a non-native trans Pro32 essential for aggregation into amyloid (Jahn et al., 2006), and possess a re-packed hydrophobic core as a consequence of the loss of the N-terminal six amino acids (Figure 1; Eichner et al., 2011). These unique features of the ΔN 6 amyloid precursor imply specificity in the early stages of assembly, in that this species, and no other, more highly unfolded states is the most amyloidogenic species in the folding energy landscape (Karamanos et al., 2016). For β_2_m, there is no simple relationship between thermodynamic stability and amyloid aggregation, as exemplified by the murine protein, mβ_2_m, which is less stable than ΔN 6, yet does not readily aggregate into amyloid, at least under most conditions *in vitro* (Karamanos et al., 2016). An interesting property of this system is that the interaction of the ΔN 6, hβ_2_m and mβ_2_m variants in different combinations has different effects on the timecourse of aggregation, with the ΔN 6-mβ_2_m interaction inhibiting the aggregation of ΔN 6, while the ΔN 6-hβ_2_m interaction promotes the self-assembly of hβ_2_m (Karamanos et al., 2014). The affinities of both complexes are low (K_d_ ∼50 and 500 μM, respectively), yet clear evidence for a 1:1 interaction between the proteins can be detected by NMR chemical shift perturbation and by NMR PRE studies (Karamanos et al., 2014). Using this information, ensembles were generated using intermolecular PRE values that describe the association of these protein pairs in a quantitative manner using simulated annealing docking calculations as shown in Figure 5A (Karamanos et al., 2014). The resulting ensembles showed that although similar parts of the proteins involving the loops surrounding the important trans Pro32, are involved in both interfaces, the structural ensembles are distinct: the interface for the inhibitory ΔN 6-mβ_2_m interaction is less diffuse than that of the ΔN 6-hβ_2_m complex and involves more hydrophobic interactions than its amyloid-competent counterpart.

The visualization of these transient interactions is not only a neat biophysical experiment that demonstrated a surprising specificity to the transient ensembles that drive or inhibit amyloid assembly, but it also led to the development of new strategies to inhibit assembly of ΔN 6, by targeting the early protein-protein interactions that drive assembly (Cawood et al., 2020). Specifically, by taking advantage of the interfaces identified, screening for a small molecule inhibitor of assembly was performed using disulfide tethering, in which a unique Cys was placed in the interface of interest and a library of small molecules (each as a symmetrical disulfide) was screened using ESI-MS (Cawood et al., 2020). The result was a fragment that covalently binds to the interface region and inhibits assembly by stabilizing an off-pathway tetramer (Cawood et al., 2020; Figure 5B). Remarkably, the ligand-bound tetramer was crystallized, providing an atomic-level view of a trapped oligomer and a complete understanding of why this structure is incompatible with the on-pathway dimer fold (Figure 5B). This finding opens up opportunities to target heterogenous/transient interactions that are normally considered undruggable in these dynamic proteins, since the covalent tethering approach is generic, does not require prior structural information and the proteins involved lack a well-defined pocket. This contrasts with the design principles of tafamidis that inhibits the aggregation of transthyretin and is now in clinical use (Ruberg et al., 2019).

## Conclusion

Dynamic protein states such as those involved in protein misfolding and aggregation represent a challenge to structurally characterize using X-ray crystallography and cryo-EM. Generating realistic representations of these dynamic protein systems requires measurement of a plethora of restraints using an array of experimental methods that report on long- and short-range interactions. Detailed understanding and appreciation of how the timescale of protein conformational exchange affects the interpretation of the experimental data is needed to generate restraints that realistically describe the experimental parameters. However, when these restraints are properly averaged to reflect the time averaging of events occurring in solution, detailed structural ensembles can be generated. Clustering of these ensembles using powerful machine learning techniques holds promise in understanding the structural propensities that cause only a few of these molecules to self-assemble to pathological aggregates and why other disordered species are aggregation-resilient. With the progress in machine learning, combined with proper treatment of experimental restraints, we may soon be able to visualize dynamic protein ensembles in intricate detail and pick out individual conformers able to drive or arrest protein aggregation, including the downhill cascade into amyloid fibrils.

## Author Contributions

TKK wrote the first draft. TKK, APK, and SER contributed to manuscript revision and conceptualization, read, and approved the submitted version. All authors contributed to the article and approved the submitted version.

## Conflict of Interest

The authors declare that the research was conducted in the absence of any commercial or financial relationships that could be construed as a potential conflict of interest.

## Publisher’s Note

All claims expressed in this article are solely those of the authors and do not necessarily represent those of their affiliated organizations, or those of the publisher, the editors and the reviewers. Any product that may be evaluated in this article, or claim that may be made by its manufacturer, is not guaranteed or endorsed by the publisher.
